# Biological Sources of Intrinsic and Extrinsic Noise in *cI* Expression of Lysogenic Phage Lambda

**DOI:** 10.1038/srep13597

**Published:** 2015-09-02

**Authors:** Xue Lei, Wei Tian, Hongyuan Zhu, Tianqi Chen, Ping Ao

**Affiliations:** 1 Shanghai Center for Systems Biomedicine, Shanghai Jiao Tong University, Shanghai, P. R. China; 2 Department of Bioengineering, University of Illinois at Chicago, Chicago, IL, USA; 3 Department of Computer Science and Engineering, University of Washington, Seattle, WA, USA; 4 State Key Laboratory for Oncogenes and Related Genes, Shanghai Cancer Institute, Shanghai Jiao Tong University School of Medicine, Shanghai, P. R. China

## Abstract

Genetically identical cells exposed to homogeneous environment can show remarkable phenotypic difference. To predict how phenotype is shaped, understanding of how each factor contributes is required. During gene expression processes, noise could arise either intrinsically in biochemical processes of gene expression or extrinsically from other cellular processes such as cell growth. In this work, important noise sources in gene expression of phage λ lysogen are quantified using models described by stochastic differential equations (SDEs). Results show that DNA looping has sophisticated impacts on gene expression noise: When DNA looping provides autorepression, like in wild type, it reduces noise in the system; When the autorepression is defected as it is in certain mutants, DNA looping increases expression noise. We also study how each gene operator affects the expression noise by changing the binding affinity between the gene and the transcription factor systematically. We find that the system shows extraordinarily large noise when the binding affinity is in certain range, which changes the system from monostable to bistable. In addition, we find that cell growth causes non-negligible noise, which increases with gene expression level. Quantification of noise and identification of new noise sources will provide deeper understanding on how stochasticity impacts phenotype.

Phenotypes of organisms, which are thought to be shaped by their genotypes and environments, often show large variation even when there is no genotypic or observable environmental difference^1^,^2^,^3^,^4^. This inherent stochasticity is ubiquitous in biological processes, such as development and disease^5^. Usually, cells tend to keep gene expression noise low enough to maintain relatively stable states. On the other hand, when environmental and/or cellular conditions change, cells can change to states of higher fitness by the aid of noise^6^. It is challenging to understand these paradoxical effects of noise in cells. Moreover, noise arises in one single component can usually influence the whole system in some unpredictable way^7^, making the problem even difficult. In order to examine how noise affects biological processes, how cells keep noise that arises intracellularly and/or extracellularly in control, and how cells are even able to exploit noise, understanding of where noise comes and quantification of noise effects are required in the first place. Among related fields, noise effects on gene expression have drawn most attention since gene expression is central to almost all cellular functions, and is vulnerable to fluctuation, owing to the low copy number of genes and their transcripts^8^,^9^,^10^. Noise that affects gene expression is usually classified into categories: intrinsic noise and extrinsic noise^11^. Intrinsic noise is defined as the stochasticity of biochemical interaction of particles in gene expression process, while extrinsic noise is generated from other cellular processes or from environmental fluctuation.

In this work, phage λ is used to study gene expression noise. After infection into its host *Escherichia coli*, phage λ makes a decision between two modes of growth, lysis and lysogeny. In the lytic mode, phage λ generates a large number of progeny and lyses the cell, while in the lysogenic mode, phage λ integrates its DNA into host genome and keeps latent along with growth and division of its host for generations. Protein CI, product of gene *cI,* plays a crucial role in maintaining lysogenic state. Phage λ lysogenic state is quite stable without induction^12^. Spontaneous switching rate from lysogenic state to lytic state is less than 10^−8^ per generation^13^, which is expected to be the result of noise in gene regulation by CI^14^. However, *cI* expression in lysogenic phage λ is not strictly constrained in a small range. Instead, experimental data show that there is remarkable variation among lysogen population^15^. This variation represents the expression noise of gene *cI*.

Gene expression noise in *E. coli* has been widely studied, which gave quantifications of intrinsic and extrinsic noise of dynamics and of steady states, from single cell to population^11^,^16^,^17^,^18^,^19^, revealing a number of factors that affect gene expression noise in *E. coli*, including gene regulation, fluctuation of transcription rate^11^ and of protein production rate^19^. These factors could also play a role in phage λ system. Besides, DNA looping was reported to have important impacts in maintaining phage λ lysogenic state^20^. Effect of DNA looping on noise has been studied in the *lac* system, which has two operators, fewer than the six operators in phage λ system. However, the results from *lac* system are sort of controversial: noise could be either increased^21^ or decreased^22^. In phage λ system, DNA looping effects on noise is still unclear. Apart from the intrinsic noise mentioned, cell growth, as an extrinsic noise source, could contribute to noise as well by changing cell volume and causing fluctuation of cellular particles. There are also other noise sources such as locations of molecules, DNA supercoiling, etc. In this work, we incorporated noise factors in a computational model of phage λ lysogeny system and quantified the contribution of each factor to total noise in steady-state distribution. We consider noise comes from gene regulation, transcription, and translation, and are able to explain a considerable fraction of noise observed in a previous experiment^15^. The explained noise according to where they come can be further divided into explained intrinsic noise (from gene regulation, transcription and translation) and explained extrinsic noise (from cell growth).


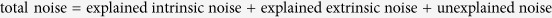


From view of modeling, several models have been developed, focusing on noise sources and their mathematical representations^23^,^24^,^25^. However, modeling noise in phage λ is challenging, since there are six operators in the regulation region of *cI* expression, resulting a state space too huge to be solved using these models. In this work, we use SDEs, which are convenient to study noise owing to the separation of deterministic terms and stochastic ones in their mathematical structure, as well as their good scalability to systems with large state space. The description of promoter state space follows the physico-chemical model used by Shea and Ackers^26^. Although this model takes the assumption of quasi-equilibrium, which means that the rate constant of transcription factor binding and/or unbinding its relative operator is large to get fast equilibrium, it is enough to model average situations as population heterogeneity in steady states. We will show that the model is appropriate in exploring the contribution of each noise source. Factors like memory and bursting, which are important noise sources affect gene expression dynamics^25^,^27^,^28^, are not included in the model, since these noise in dynamics will be averaged out for proteins with long enough lifetime^18^, such as protein CI, which barely degrades in normal experimental conditions.

In this work, we will address two questions: (1) How to quantify intrinsic and extrinsic noise from fundamental mechanism of *cI* expression in phage λ lysogen. We focus on the population fluctuation from an ensemble view and give the mathematical definition of each noise. (2) How and how significantly each noise factor affects gene expression.

## Results

### Mathematical definition for deterministic terms of SDE

In our previous work^4^ on quantifying noise of *cI* expression with chemical Langevin equations (CLEs)^29^, we simplified the expression processes by integrating transcription and translation into one virtual process, resulting in a system the state of which can be described by the birth-death process of protein CI. We refer to this model as one-step expression model here. Steady-state distribution of CI was obtained via a potential construction method^30^,^31^,^32^. The results of mean expression levels agreed quite well with experiments^4^. However, the noise explained by the one-step expression model was much smaller than that observed in experiments^15^, implying that effects of mRNA fluctuations might be overlooked due to the one-step simplification. A previous study showed that multiple biochemical steps of gene expression can be well approximated by simplified synthesis and degradation of mRNA and protein when quantifying gene expression noise^24^. In this paper, we present a model considering noise from mRNA fluctuation in addition to that from protein fluctuation, which we refer to as two-step expression model. Processes involved in *cI* expression can be written as the reactions below.


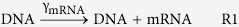



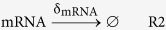



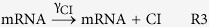



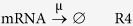



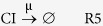


γ_mRNA_ and γ_CI_ are the transcription initiation rate and CI synthesis rate, respectively. δ_mRNA_ is the degradation rate of mRNA. Since CI is quite stable and barely degrades, it is assumed only to be diluted because of cell growth, where μ is the dilution rate. The parameters and their corresponding references are listed in Table 1. The system can be described by following equations:


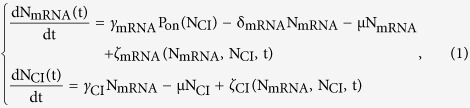


where N_mRNA_ and N_CI_ are the total numbers of mRNA and of protein CI, respectively. ζ is the noise term including both intrinsic and extrinsic noise which we will describe in detail later. P_on_ denotes the probability that P_RM_, the promoter of *cI*, is in the “on” state, i.e. P_RM_ is vacancy for RNA polymerase to bind to.

P_on_ characterizes the regulation of *cI* expression, which depends on how protein CI binds to the gene operators OR and OL. Each operator contains 3 binding sites for free CI dimers with the binding affinity OR1 > OR2 > OR3 and OL1 > OL3 > OL2^15^,^26^,^33^ (Table 2). Two CI dimers bound at adjacent binding sites, such as OR1 and OR2, can form a tetramer. Two CI tetramers on OR and OL can further form an octamer, resulting a DNA loop. As the distance between OR and OL is as long as 2.3 Kb, it is possible for the loop to form between DNA in either same or opposite orientations. All these combinations give 113 possible binding configurations between CI and operators in total, 32 of which are looped^15^,^33^ (Supplementary Table S1). The probability of the *i*-th binding configuration P_i_ follows a grand canonical distribution^26^,


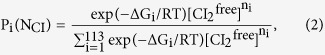


where ΔG_i_ is the free energy of the *i*-th binding configuration that can be calculated from well-accepted biochemical experiments^34^,^35^ (Table 2). R is gas constant, and T = 310 K is absolute temperature of experimental conditions^15^. n_i_ is the number of CI dimers bound to the operators. With the probability of each binding configuration, we can calculate the synthesis rate of mRNA,


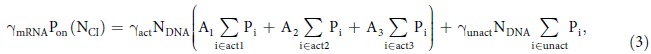


where N_DNA_ is the average number of DNA molecules in a *E. coli* cell. γ_act_ and γ_unact_ are the rate constants of transcription when P_RM_ is in the active state and in the basal state, respectively. According to the predicted DNA looping mechanism^15^, all the activated configurations can be grouped into three different activation levels A1, A2, and A3. The predicted looping mechanism was recently confirmed by Cui and Murchland *et al.*^33^ with some detailed differences (Supplementary Text S1). Detailed grouping of activation states of different looping mechanisms can be found in Supplementary Table S1. Results show that different looping activation mechanisms can be distinguished by gene expression levels, while they made little difference in terms of noise (Supplementary Fig. S1).

Besides DNA looping, binding of CI dimer to the nonspecific DNA sites is also another important factor that causes *cI* expression fluctuation. Apart from OR and OL, CI can bind to other DNA sites, which affects the concentration of free CI dimers. Indeed, only a small fraction of CI dimers in the cell is free to bind specifically owing to the large size of the whole genome of host *E. coli* (4.6 Mb). The following equation shows the relationship between the concentration of total CI and that of free dimers^36^


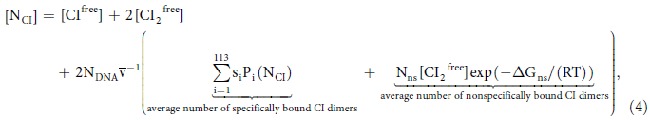


where [CI^free^] and [CI_2_^free^] are free CI monomer and dimer concentration, respectively. 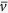 is the effective cell volume. S_i_ ∈ {0, 1, 2, …, 6} is the number of CI dimers bound to OR and OL. N_ns_ is the length of whole genome in *E. coli* and ΔG_ns_ the nonspecific binding energy of CI.

### Mathematical definition of intrinsic noise

Intrinsic noise is generated in biochemical processes of gene expression. Thus noise term ζ in Eq. (1) is defined as the intrinsic noise term ζ_in−_ in forms of Gaussian white noise which can be derived from reactions R1–3^29^.


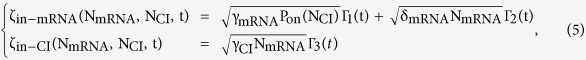


where


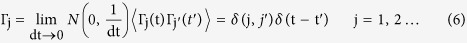


Random variable Γ follows normal distribution with mean 0 and standard deviation 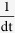. The first Delta function is Kronecker’s and the second Dirac’s. Dilution caused by cell growth is not an intrinsic noise source.

The system is described by Eqs (1), (5) and (6). We compute the steady-state distributions of five genetic constructs which were designed in previously reported experiments^15^ to study how DNA looping affects lysogeny CI expression. In our computation, the five constructs are different in their binding energies between CI proteins and gene operators. For example, the binding energy of operator OR3 in the construct OR3-r1 is different from that of WT, while all the other binding energies, as well as the kinetic parameters, are the same with WT. The binding energies can be found in Table 2. By comparing the computational results with the experimental data measured in^15^, we study the effects of different noise factors quantitatively.

**Figure 1:**
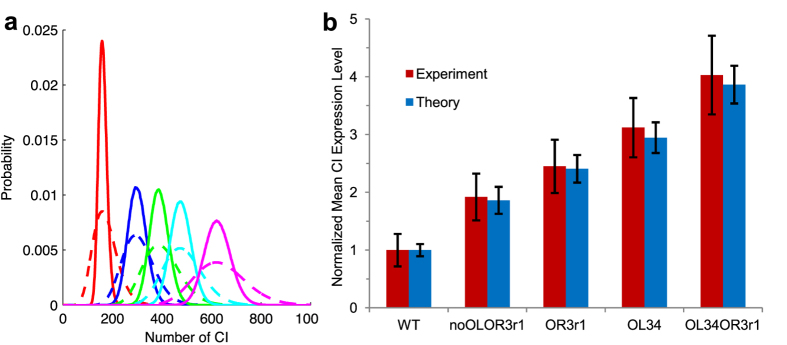
Ito simulation results of the two-step expression model. (**a**) Steady-state distribution of CI copy numbers. The solid and the dashed lines represent the computational and rescaled experimental results, respectively. Each plot was calculated with 10^10^ runs, and the error is within 10^−6^. Fluorescence intensity of experimental results is assumed to be proportional to the number of CI, thus experimental results can be compared with computational ones by rescaling the data with a corresponding ratio between modes of experimental and of computational results. (**b**) Comparison of mean CI expression level between experiments and computation. Error bars are calculated as normalized standard deviations.

Coefficient of variation (CV), calculated as standard deviation divided by mean, is used as a noise indicator, reflecting the magnitude of variability as a percentage of the level of gene expression. Percentage of explained noise is defined as CV_model_/CV_experiment_. 37–60% of the total noise from experimental results of five constructs can be explained by the two-step expression model, respectively (Supplementary Table S2). To compare mean expression level of CI between experiments and computation, mean values of different constructs are normalized by the corresponding mean value of WT (Fig. 1b). Results show that mean expression level obtained by the two-step expression model agrees well with experimental results^15^ in all five constructs.

### Hierarchy of intrinsic noise sources

**Figure 2:**
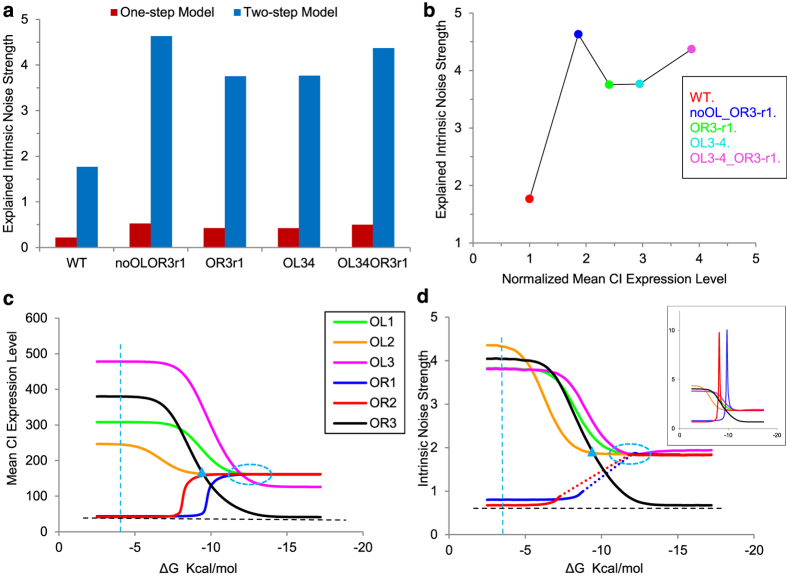
Intrinsic noise sources hierarchy and their functional roles. (**a**) Intrinsic noise strength calculated from the one-step and the two-step expression model, corresponding to level (1) noise. Results for the one-step expression model are from reference^4^. (**b**) Noise strength calculated from the two-step expression model varies with normalized mean CI expression level in five different constructs, corresponding to level (2) noise. (**c**,**d**) show level (3) noise. Mean CI expression level (**c**) and intrinsic noise strength (**d**) vary with binding energy of the corresponding operators, respectively. Notice that dashed region in OR1 and OR2 in (**d**) actually contain two peaks, details of which are shown in inset. Blue vertical dashed lines in (**c**,**d**) represent the nonspecific binding energy (−4.1 Kcal/mol). Binding energy of WT operators lies in the region circled by blue dashed ellipses. The inset represents the full figure of (**d**).

Since all kinds of factors can have impacts on system overall noise, we classify noise sources into three different hierarchical levels to make the problem clear, from directly observed particle numbers to fundamental mechanisms: (1) fluctuation of particles (including both mRNA and protein); (2) gene regulation which affects particle fluctuation; (3) CI binding energies to the gene operators that affect gene regulation. In the first level, mRNA fluctuation that was not included in the one-step expression model is considered in the two-step expression model. Comparison of noise strength between the results of the two models shows that mRNA fluctuation contributes significant part in *cI* gene expression noise (Fig. 2a). Noise strength is measured by Fano factor, which is defined as variance divided by mean^16^. In the second level, we consider more details of gene regulation that affect mRNA fluctuation which include CI autorepression and DNA looping. In five different constructs, the mutation OR3-r1 is a defect impairing P_RM_ repression, and OL3–4 prevents P_L_ from assisting P_RM_ repression. Comparison among strength of intrinsic noise of the five constructs show that autorepression decreases the intrinsic noise in WT, and deficient repression can lead to relatively high intrinsic noise (Fig. 2b WT and OR3-r1, also WT and OL3–4). Similar conclusion was obtained in an engineered autorepression loop using tetracycline repressor^37^. Loop formed by DNA and CI also counteracts part of intrinsic noise (Fig. 2b WT and noOL_OR3-r1, also noOL_OR3-r1 and OR3-r1). We construct a noOL mutant computationally, in which CI affinity with all three operators in OL is removed. Results show that intrinsic noise strength of noOL (Fano = 4.2) is higher than that of WT (Fano = 1.8). One plausible explanation is that DNA looping stabilizes the WT phage λ lysogeny system^20^. Results show that autorepression has more impacts (about two fold) than DNA looping in reducing noise (Supplementary Table S2).

In the third level, we examined how operators affect regulation noise by varying the binding energy of each operator while leaving others the same as WT, which is a computational single mutation on the corresponding DNA operator. Experimentally, this can be carried out precisely by modern biological technologies^38^. Results show that noise strength (and also expression level) of each operator changes with binding energy in a sigmoidal manner: the curve of each operator has two plateau regions (Fig. 2c,d). Binding energies of WT OL, OR1 and OR2 all lie on the right plateau, which seems to imply that impacts of these operators onto total noise already reach their limits or stable regions. Nonspecific binding energy (−4.1 Kcal/mol) is in the other stable region (blue vertical dashed lines in Fig. 2c,d). OR3 is a key operator, to which both protein expression level and noise are sensitive. Its binding energy value of WT gene type is in the middle of the changing range (yellow triangles in Fig. 2c,d).

Single mutation in WT reveals the role of each operator to expression noise. The noise strength increases with the binding energy of OR1 and OR2, while decreases with those of OL and OR3, indicating that the negative feedbacks from OL and OR3 reduce noise, while positive feedbacks from OR1 and OR2 increase noise. This agrees with the prediction in a previous study^21^. Similar results can be obtained by doing single mutation to other constructs (Supplementary Fig. S3). In construct OL3–4 and OL3–4_OR3-r1, negative feedback from OL3 is impaired, leading to the consequence that OL1 and OL2 take the role of positive feedbacks (Supplementary Fig. S3c and S3e). Intrinsic noise strength and expression level have the same changing trend when varying with binding energies in WT. High expression level corresponds to high noise, while finite numbers correspond to the order. This trend actually depends on the parameter used to change expression level and noise^16^. Besides, the lower plateaus of OR1, 2 and 3 share the same lower bounds for both mean expression level and noise level (black horizontal dashed line in Fig. 2c,d). Similar lower bounds of expression noise have been reported in a eukaryotic system^39^. Our results show that in phage λ lysogeny system, removing positive feedbacks from OR1 and/or OR2 reaches the same level as increasing negative feedbacks from OR3 to the maximum, for both mean expression levels and noise strength levels (Fig. 2c,d, Supplementary Table S3). The lower bounds represent basal expression of promoter P_RM_ and the corresponding basal expression noise. Besides, our computation shows that in the jumping regions of OR1 and OR2, there are two intriguing peaks (Fig. 2d inset). We will discuss these peaks later in the discussion section.

### Mathematical definition of extrinsic noise from cell growth

Besides intrinsic noise from biochemical processes of gene expression, fluctuations of other cellular components and processes generate extrinsic noise. Cell growth, one of extrinsic noise sources, is reported to have important impacts^40^. Cell growth dilutes the concentration of cellular particles, which causes fluctuation inside the cell. Here we extend the aforementioned model to incorporate the noise caused by the exponential cell growth using two models. In the first model, dilution is considered as a degradation reaction with a rate corresponding to the constant cell growth rate, which is a classic way to treat the dilution in a biochemical system. In the second model, we will further consider the case when cell growth rate is not a constant, which is a more realistic model than the classic one.

### Constant cell growth model

When cells are in exponential growth phase, the cell growth rate can be simplified as a constant on the logarithmic scale. The dilution of the cellular particles caused by cell growth can be described as a ‘degradation reaction’ (Reaction R4 and R5) with the reaction rate equals to the mean cell growth rate. This approach is commonly used when modeling gene expression using ODEs (ordinary differential equations)^41^, and can be easily extended to CLEs^40^,^42^. In this way, noise of cell growth comes from the stochasticity of when the degradation reaction happens. The chemical reactions in the system (R1–5) give the following CLEs, which describe the constant cell growth model.


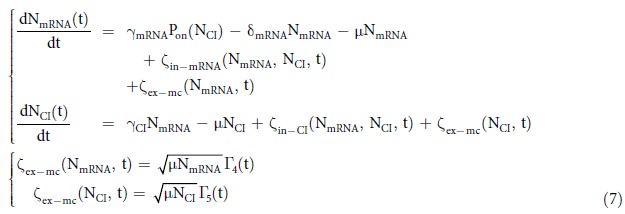


**Figure 3:**
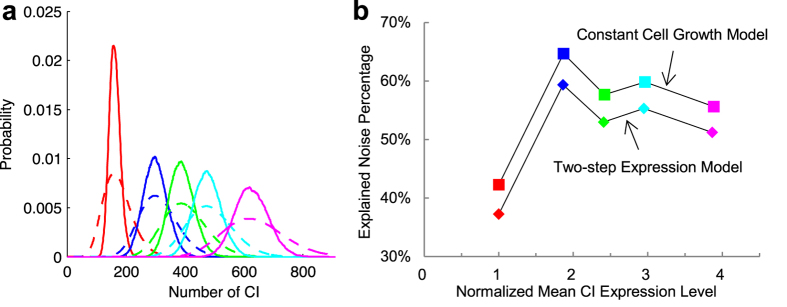
Ito simulation results of the constant cell growth model. (**a**) Steady-state distribution of CI protein, with solid and dashed lines representing computational and experimental results, respectively. (**b**) Noise comparison between the two-step expression model and the constant cell growth model for the five genetic constructs.

The intrinsic noise terms ζ_in−_ satisfy Eq. (5), and Γ satisfy Eq. (6). The subscription *ex-mc* denotes the extrinsic noise terms that come from the cell growth with constant rate. The rate value can be obtained by measuring the mean value of cell growth in experiments. We used Ito simulation to solve Eq. (7) and stochastic simulation algorithm (SSA) to simulate reactions R1–5^43^ (Supplementary Table S2). The steady-state distribution is shown in Fig. 3a.

Mean expression levels agree well with experimental results, same as the two-step expression model. In addition, more noise can be explained by this model which incorporates the stochastic effects of cell growth (Fig. 3a,b). Results (Fig. 3b) show that, extrinsic noise caused by constant cell growth increases a small amount (4–5%) in all five constructs compared to the two-step expression model. A method was developed^44^,^45^ to calculate the contribution of each reaction to the overall noise of a biochemical system when the reactions of the system are Poisson processes. We apply this method on our constant cell growth model, and the results agree with ours (Supplementary Fig. S4).

### Stochastic cell growth model

Compared to being a constant, cell growth rate is more likely to vary over time (stochastic cell growth rate). In addition to the mean cell growth rate, cellular particle concentration is further influenced by the variation of the growth rate. In this section, we will first derive the noise from this variation, and then extend the constant cell growth model to incorporate this noise, which will give us a whole picture of how cell growth affects noise in gene expression. By introducing a stochastic term into the classic exponential growth equation V(t) = V(0)exp(μt), the variation of the cell growth rate can be considered,


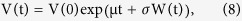


where V(t) is the average volume of the cell population at time t, μ is the cell growth rate, and σ is the stochastic factor. W(t) is a Wiener process.

The gene expression noise arises from the variation of the cell growth rate is (see Supplementary Text S2 for detail derivation)


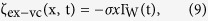


the subscription ex-vc denotes that this extrinsic noise is caused by the variation of the cell growth rate. Since the noise term ζ_ex−mc_ comes from mean of the cell growth rate, and the noise term ζ_ex−vc_ comes from the variation, we can incorporate this noise into the constant cell growth model to get the stochastic cell growth model with no double-counting.


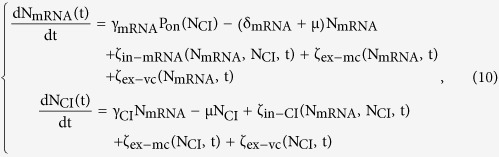


where


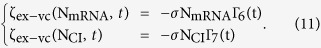


Due to the stochastic cell growth rate, cell cycle varies with the time intervals between consecutive divisions^46^. The value of σ can be estimated from the variation of cell doubling time (Supplementary Text S2),


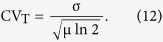


**Figure 4:**
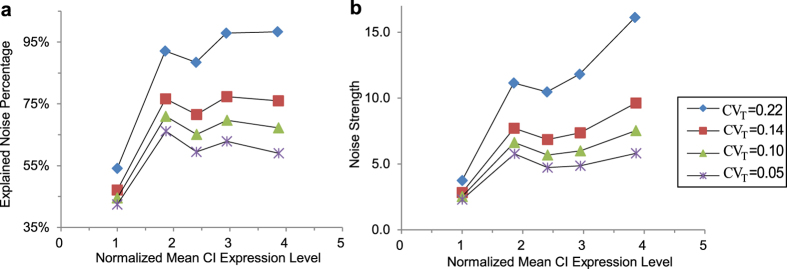
Ito simulation results of the stochastic cell growth model. Total explained noise percentage (**a**) and total noise strength (**b**) for different CV_T_. 0.22 is calculated from the *E. coli* system^19^ with the cell cycle period 45 ± 10 min. 0.14 is calculated with CV of cell growth rate 0.13 in system^40^ using the method in^40^. 0.05 and 0.10 are estimated from cell doubling time in^17^.

Since CV_T_ of five constructs were not measured, we use the values reported in other works with experimental conditions similar to^15^. Being a global parameter, we assume that CV_T_ is same for all the five constructs. The extrinsic noise term in Eq. (10) is unbiased, so the results of the stochastic cell growth model show the same expression levels as those of Fig. 1b above. The portion of explained noise in total noise is represented in Fig. 4a. The results show that noise from cell growth increases with CV_T_. When CV_T_ reaches 0.22, which means the cell doubling time is ~26 ± 5.7 min, 98% of total observation noise in OL3–4_OR3-r1 can be explained. If cell doubling time is 26 ± 3.6 min (CV_T_ = 0.14), extrinsic noise caused by cell growth contributes about 10% to total noise in WT, which has the smallest mean expression level among the five constructs, and contributes about 25% in OL3–4_OR3-r1, which has the largest mean expression level. The results show that noise from cell growth, including the effects from both the mean and the variation of growth rate, increases with expression levels (correlation coefficient 0.965). It was reported that gene expression noise in *E. coli* follows some global constraints, resulting in a positive correlation between gene expression levels and noise^47^. Our results show that one of this global constraint is cell growth.

## Discussion

**Figure 5:**
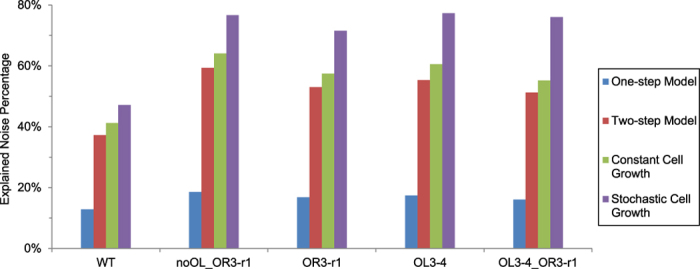
Comparison of noise percentage explained by four models. Results of the stochastic cell growth model using CV_T_ = 0.14^40^.

We compared explained noise percentage of all four models (Fig. 5, Supplementary Table S2). The one-step expression model, which does not model mRNA explicitly, explained 13–18% of total noise observed in experiments. The two-step expression model, which incorporates mRNA and models both transcription and translation explicitly, explained 37–60% of total observation noise. Comparison of these two models implies that the fluctuation of mRNA contributes the major part of intrinsic noise. For extrinsic noise, we considered noise coming from cell growth. If cell growth rate is a constant (constant cell growth model), which is usually not the case, cell growth contributes 4–5% to the total observation noise. When the variation of cell growth rate is also considered, the stochastic growth model showed that it can have large impacts on extrinsic noise. For example, when CV_T_ is 0.14, the model shows that 10–25% of total observed noise comes from cell growth. In WT, both intrinsic and extrinsic noise explained by the modeled mechanisms (about 37% and 10% respectively) is remarkably smaller compared to that in the other constructs (about 55% and 20% respectively), which implies that there might be some other mechanism not considered in this work affects WT more than the others. Some fraction of noise cannot be explained for all five constructs, suggesting there are still other unincorporated intrinsic and extrinsic noise sources.

**Figure 6:**
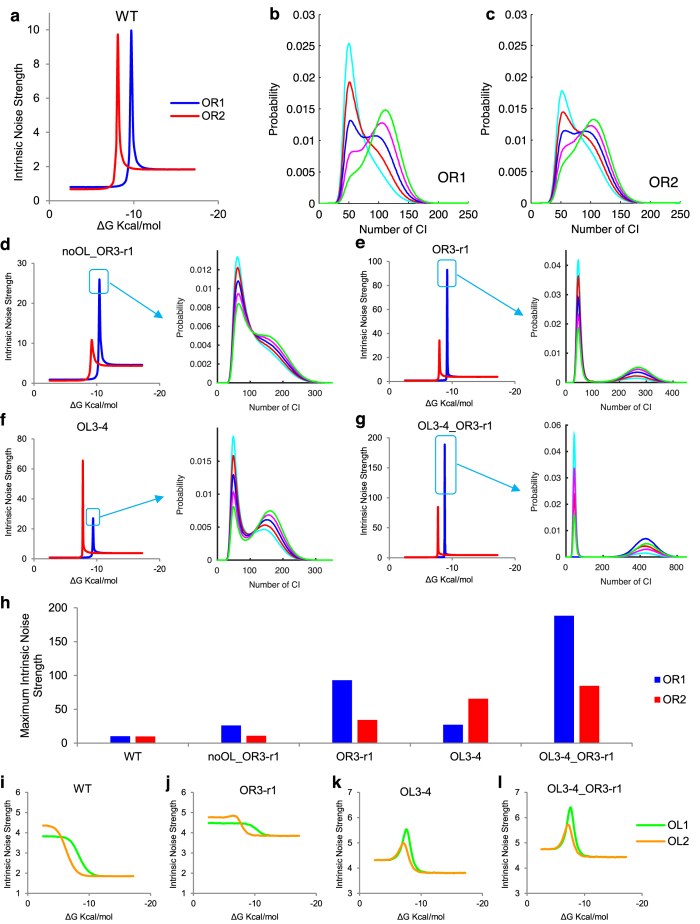
Intrinsic noise strength varies with binding energy of positive feedback operators. (**a**) Intrinsic noise strength varying with binding energy of OR1 or OR2 in WT. Steady-state distribution of WT *cI* expression changes with binding energies of OR1 in (**b**) and OR2 in (**c**). Binding energies from low to high are in color cyan, red, blue, magenta and green. In (**b**), the binding energies of OR1 are −9.64, −9.68, −9.72, −9.76 and −9.80 Kcal/mol for the curves in corresponding colors, respectively. In (**c**), the binding energies of OR2 are −8.07, −8.09, −8.11, −8.13 and −8.15 Kcal/mol. (**d**–**g**) Peaks in the other four constructs when vary binding energy of OR1 or OR2, and their corresponding steady-state distribution with binding energy around the peaks for OR1. A whole set of figures of bimodal steady-state distribution for both OR1 and OR2 in the four genetic constructs is in Supplementary Fig. S5. (**h**) Height of peaks in five constructs. (**i**–**l**) Intrinsic noise strength varies with binding energy of OL1 or OL2. Each point is calculated from 10^8^ Ito simulation runs using the two-step expression model, the errors of which are within 10^−6^. The increasing step of binding energy is 0.3 kcal/mol in plateau regions and 0.01 kcal/mol in peak regions.

When studying how each operator affects noise in the system, we found intriguing peaks in noise-binding-energy curve (Figs 2d and 6a). Why binding energy in that region gives rise to those two high noise peaks still needs to be discussed. For operator OR1 and OR2, how intrinsic noise strength changes with binding energy is remarkably different from that of the other operators. The noise strength curves of OR1 and OR2 show sharp peaks between the left and the right plateaus (Fig. 6a), other than the common sigmoid pattern of the other operators (Fig. 2d). When the binding energy of the operator falls in the left (right) plateau, the steady-state distribution of the system is unimodal and shows a low (high) expression state. As the binding energy increases, system intrinsic noise strength increases as well. When the binding energy comes to the peak region, the steady-state distribution of the system shows an unusual bimodal shape, which indicates a bi-expression state of the system (Fig. 6b,c). The two peaks of the bimodal distributions become more separated in the other four genetic constructs (Fig. 6d–g). For all the constructs that show two peaks in their steady-state distribution, the first peaks are in the similar position, indicating a basal expression state. Operators and TF, and the cooperation among them form the positive feedback loop of the system. The positive feedback increases with the binding energy, which generally raises noise strength as we showed above. Our results show that this noise strength can be much larger during transition than that out of transition, which can raise bistability of the system. Similar bistability was observed in experiments^42^ by varying degradation rate of TF, though no noise peak was found in bistable regime but a regular sigmoid transition. A possible explanation is that we used different ways to change positive feedback. Actually, our results show that bistability only happens in those cases when sharp and high peaks exist in the noise-binding-energy curve. When the noise-binding-energy curves are sigmoidal (Fig. 2d, Supplementary Fig. S3b, S3d and S3f), bistability is not found. Besides OR1 and OR2, OL1 and OL2 also provide positive feedback in the construct OL3–4 and OL3–4_OR3-r1 according to their expression curves (Supplementary Fig. S3c and S3e). When varying the binding energies of OL1 and OL2 in these two constructs, we also find noise peaks in their noise-binding-energy curves (Fig. 6k,l). However, as the noise peaks are not sharp or high enough, which means the noise is not sufficiently strong, bistability is not found. All these indicate that when noise strength from positive feedback is large enough, expression level can be turned from monostable states to bistable states.

When varying the binding energy of OR2 in the construct noOL_OR3-r1, the noise-binding-energy curve shows the maximum intrinsic noise strength is 10.8, which is close to that of WT (9.8). So, we expected a bimodal-shaped steady-state distribution of noOL_OR3-r1, just like WT. However, results only show a distribution with a unimodal shape (Supplementary Fig. S5). Interestingly, when varying the binding energy of OR1 in the same construct, system does become unstable because of the noise, but the bimodal shapes are not as clear as those in the other constructs (Fig. 6d), although it shows a relatively large noise (26.0) in the noise-binding-energy curve. The difference between the construct noOL_OR3-r1 and the others is that noOL_OR3-r1 cannot form DNA looping due to the defective OL, implying DNA looping may destabilize the system in certain situation. This is consist with our finding that the looping increases noise when it losses the function of autorepression. Further experiments will be helpful in clarifying how system stability is related to DNA looping. Comparison among peaks of different constructs actually shows the competition between positive and negative feedbacks from the six operator-binding-sites in phage λ lysogeny system. From construct WT to OR3-r1 and OL3–4 to OL3–4_OR3-r1, negative feedbacks gradually decrease, while the maximum intrinsic noise strength increases in the same order, which implies that positive feedbacks increase when negative feedbacks decrease (Fig. 6d–h).

In summary, this work contributes from two perspectives. Firstly, we quantify the effects of several noise sources of *cI* expression in lysogenic phage λ in one mathematical framework. Our results show that transcription and translation contribute to ~50% noise in steady states. We show that in phage λ system autorepression counteracts noise and autoactivation increases noise. For DNA looping, which plays a subtle role in CI autoregulation since either positive or negative feedback is possible, our computational results show that it reduces noise in WT phage λ lysogeny system, whereas may increase noise in mutants autorepression of which is impaired. Besides, intriguing noise peaks arise when varying binding energy of OR1 and OR2. These peaks change the system from monostable to bistable. Secondly, we propose a model to quantify extrinsic noise caused by stochastic cell growth. Results show that noise from cell growth increases with mean expression levels. Our method provides a way to study noise from fundamental biological mechanism instead of curve fitting the noise. The findings in this work give us a systematic and hierarchical understanding of how gene expression noise rises from gene regulation processes, which may help in bettering cell state maintenance or induction as well as more delicate genetic design in synthetic biology. Besides, the method, which we develop to quantify noise from cell growth, provides insight in controlling system noise of experiments, by varying the experimental conditions that affect cell doubling time.

## Additional Information

**How to cite this article**: Lei, X. *et al.* Biological Sources of Intrinsic and Extrinsic Noise in *cI* Expression of Lysogenic Phage Lambda. *Sci. Rep.* **5**, 13597; doi: 10.1038/srep13597 (2015).

## Supplementary Material

Supplementary Information

## Figures and Tables

**Table 1:** Parameters used in models.

Parameter	Value	Parameter	Value
γ_act_	^a^0.0083/s	δ_mRNA_	^a^0.0029/s
γ_unact_	^c^0.00075/s	N_DNA_	^b^3.3
γ_CI_	^ab^0.0158/s	μ	^b^0.00044/s
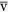	^b^2um^3^	N_ns_	^b^4.64×10^6^

^a^is from^48^, ^b^is from^15^, and ^c^is from^26^.

**Table 2:** Binding free energy used in all models follows^15^.

Parameter	ΔG(Kcal/mol)	Parameter	ΔG(Kcal/mol)
ΔGR1^a^	−12.5	ΔGL1^a^	−13.0
ΔGR2^a^	−10.5	ΔGL2^a^	−11.2
ΔGR3^a^	−9.5	ΔGL3^a^	−12.0
ΔGR3−r1^a^	−6.6	ΔGL3−4^a^	−4.1
ΔGR12^b^	−2.7	ΔGL12^b^	−2.7
ΔGR23^b^	−2.9	ΔGL23^b^	−2.0
ΔG_ns_	−4.1	ΔGCI^c^	−11.0
ΔGtet^d^	−3.0	ΔGoct^e^	−0.5

^a^is the binding free energy between CI dimer and corresponding operators. ^b^is the cooperative energy when CI dimer bound to different operator sites. ^c^is the binding energy between two monomers to form a dimer, ^d^between two dimers to form a tetramer, and ^e^between two tetramers to form an octamer.
