# Supplementary material for: Biological Sources of Intrinsic and Extrinsic Noise in cI Expression of Lysogenic Phage Lambda
Source: Sci Rep. 2015 Sep 2;5:13597. doi: 10.1038/srep13597 (PMC4557085; doi:10.1038/srep13597)
Supplement: Supplementary Information [file srep13597-s1.pdf]

Biological Sources of Intrinsic and Extrinsic Noise in cI Expression of Lysogenic Phage Lambda

Xue Lei. Wei Tian. Hongyuan Zhu. Tianqi Chen. Ping Ao.

Supplementary Information

Text S1

Different looping activation mechanisms make no difference in terms of noise.

Anderson and Yang reported that DNA looping in phage λ lysogenic system activates the gene *cI* expression and proposed two possible looping mechanism. One is called looped octamer activation model (AY's Looped Octamer Model), which assumes that the additional octamer involving conformational changes leads to enhanced activation of RNA polymerase by CI. The other one is upstream promoter (UP) element activation model (AY's UP Model), which assumes there exists a UP element around OL3 accessible to the α subunit of RNA polymerase in looped configurations, increasing promoter strength. Cui and Murchland et al recently confirmed the UP element idea by experiments and propose another UP element model (CM's UP Model) with some details different from the one of Anderson and Yang's. Cui and Murchland et al showed that UP element can activate P_{RM} only when OL3 is unoccupied regardless of the DNA looping orientation, while in AY's UP Model, UP element only activates P_{RM} in one looping orientation, unaffected by OL3 occupation.

In terms of modeling, these three models mathematically only differ in how each binding configuration is grouped and how large is the activation level of each group (acti and A_i in Equation (3)). We tried all three to examine the effects of DNA looping on system noise. Results show that there is no obvious distinction among them in term of mean CI expression level except when binding energy of OL1 is being varied. As for noise strength, results of the three mechanisms are almost the same.

When binding energy of OL1 is changed to as same as that of a NSB site (dashed

blue lines in Figure S1, -4.1 Kcal/mol), mean CI expression level is different in the three models. Explanation comes from different regulation mechanisms. AY's Looped Octamer Model is actually symmetrical for OL1 and OL3, thus mutating OL1 reaches the same expression level as mutating OL3. AY's UP Model assumes that looping activates P_{RM} even when OL1 is muted. CM's UP Model shows that looping is a little inhibitive to P_{RM} when UP element is not working. Results from mutating OL1 represent differences in mechanisms in three models. As for noise, results show that it is independent of looping activation mechanisms in three different models.

Text S2

Derivation on the stochastic cell growth model

SDE when considering stochastic cell growth only

Let V(t) be the average volume of the cell population at time t, μ is the cell growth rate and σ is the stochastic factor, $W(t)$ refers to Wiener process.

$$V(t) = V(0)\exp(\mu t + \sigma W(t))$$

Take the assumption that total particle numbers are invariable in time period dt and x(t) refers to particle concentration here,

$$x(t) \cdot V(t) = x(t+dt) \cdot V(t+dt)$$

To show how the average volume of the cell population changes after small time step dt,

$$\begin{aligned} V(t+dt) &= V(0)\exp[\mu(t+dt) + \sigma W(t+dt)] \\ &= V(t)\exp(\mu dt + \sigma dW(t)) \end{aligned}$$

Then we have

$$\begin{aligned} dx(t) &= x(t+dt) - x(t) \\ &= x(t)[\frac{V(t)}{V(t+dt)} - 1] \\ &= x(t)[\exp(-\mu dt - \sigma dW(t)) - 1] \\ &= -\mu x(t)\, dt - \sigma\, x(t) dW(t) + o(dt) \end{aligned}$$

Thus, we get the stochastic term which represents the noise generated by the variation of cell growth rate:

$$\zeta_\sigma = -\sigma\, x(t)\Gamma_W(t)$$
$$\Gamma_W(t) \sim N(0, \frac{1}{dt})$$

Parameter estimation

In order to estimate parameter μ and σ with the known cell cycle mean value, we make the following derivation.

Let

$$Y(t) = \mu t + \sigma W(t)$$

Cell cycle T is the first arrival time of Y with constant barrier ln2.

$$T = \min\{t \mid Y(t) = \ln 2\}$$

Then the mean and variance of T is get [1]

$$\langle T \rangle = \frac{\ln 2}{\mu}$$

$$VAR(T) = \frac{\sigma^2 \ln 2}{\mu^3}$$

Thus CV of cell cycle T is obtained

$$cv_T = \frac{\sigma}{\sqrt{\mu \ln 2}}$$

Reference

1. Tuckwell HC, Wan FYM (1984) First-passage time of Markov processes to moving barriers. J Appl Prob 21: 695709.

Figure S1. Comparison between different looping activation models. Mean CI expression level (a) and Intrinsic noise strength (b) varies with binding energy of protein CI to OL1. $\Delta GL1$ is the binding free energy of protein CI to OL1. Dashed blue lines show the nonspecific binding energy, -4.1 Kcal/mol. Red diamond shows the WT binding energy -13.0 Kcal/mol.

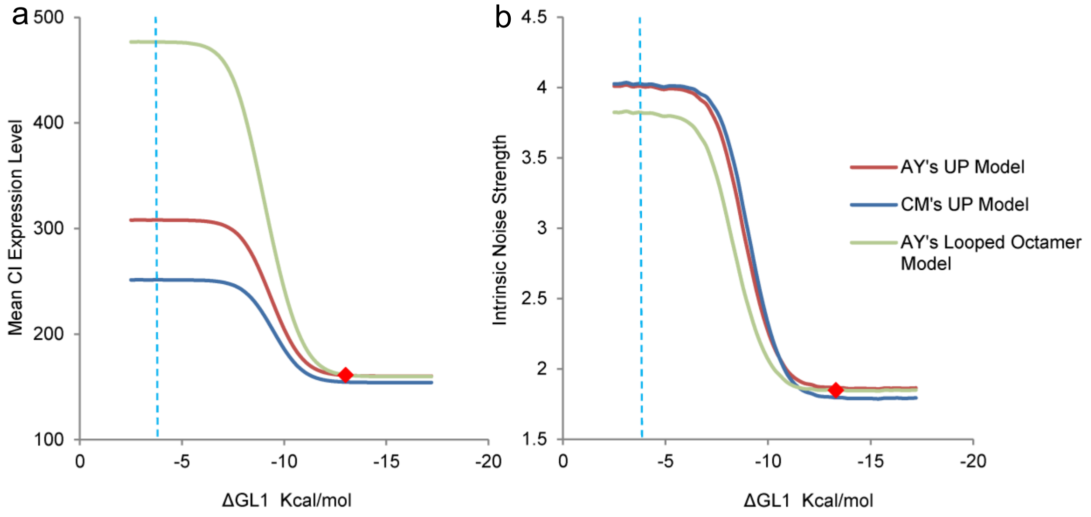

Figure S2. Comparison between AY's and CM's UP element model by varying binding energies. Results are from the Two-step Expression Model. Blue lines are results of AY's UP element activation model, and red lines CM's model. The up two columns represent mean expression level in stable states changing with binding energies (Kcal/mol). The following two columns represent noise strength. Except for OL1 mean expression level, all the other results keep the same for two models.

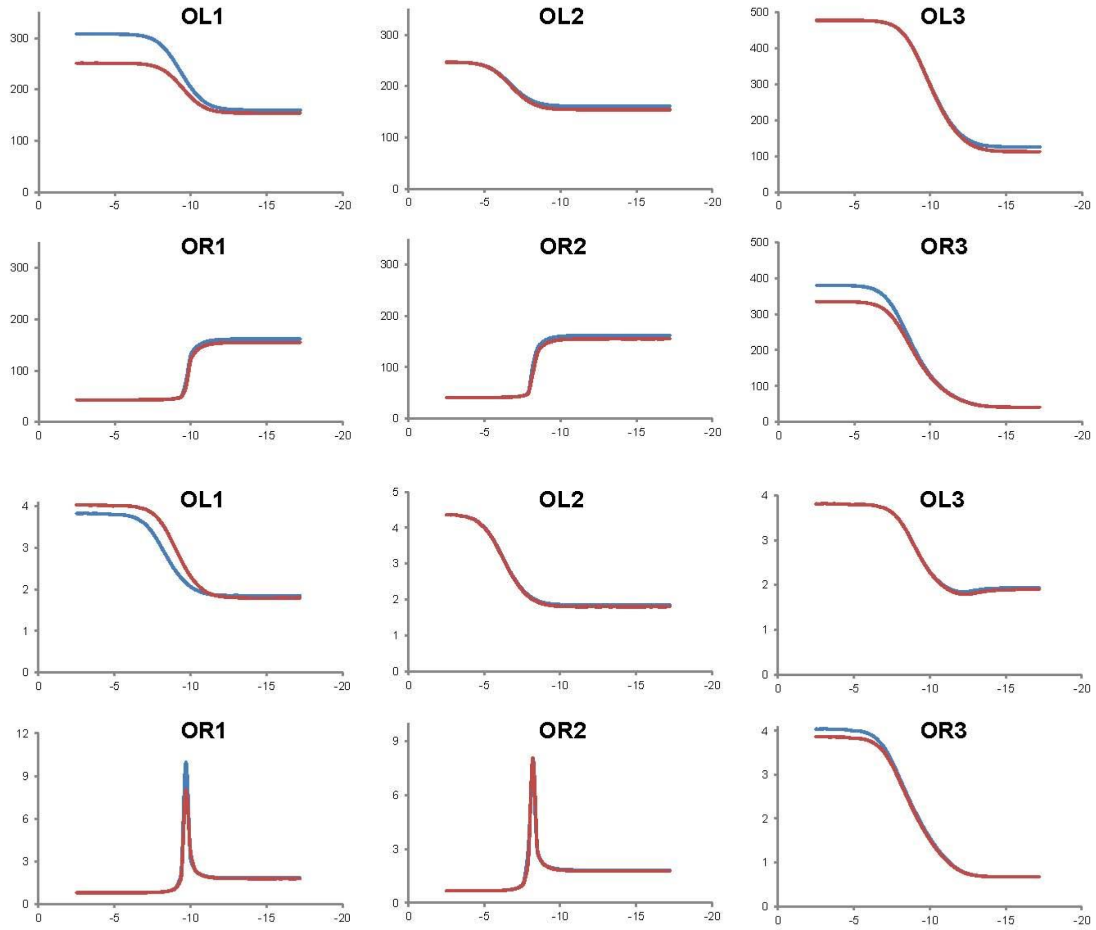

Figure S3. Contribution of each operator to expression level and noise in each construct.

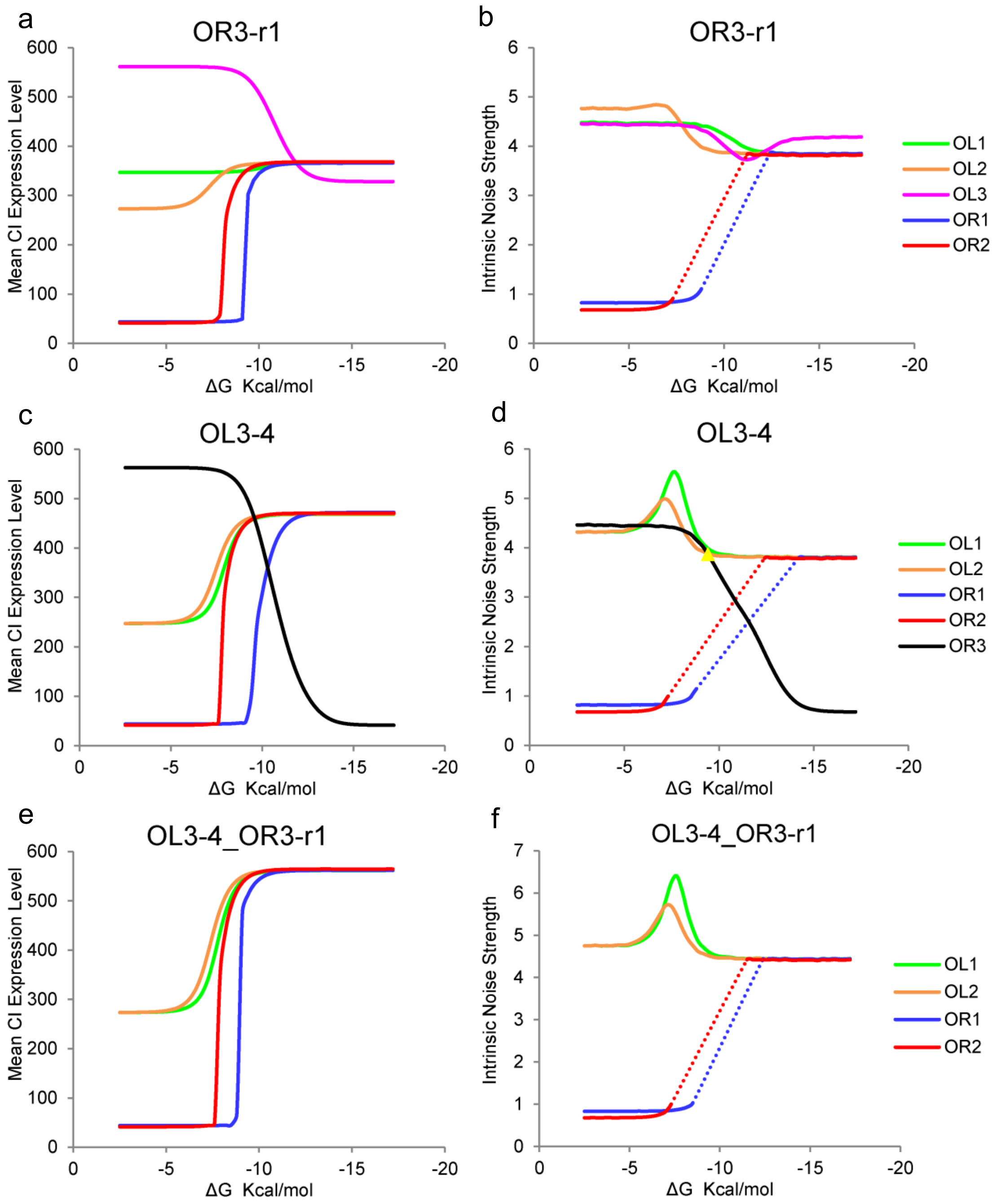

Figure S4. Noise decomposition for the constant cell growth model in WT. The framework is from Komorowski et al. 2013 and Jetka et al. 2014. Reactions 1-5 are in the order of RNA synthesis, RNA degradation, RNA dilution, CI synthesis and CI dilution.

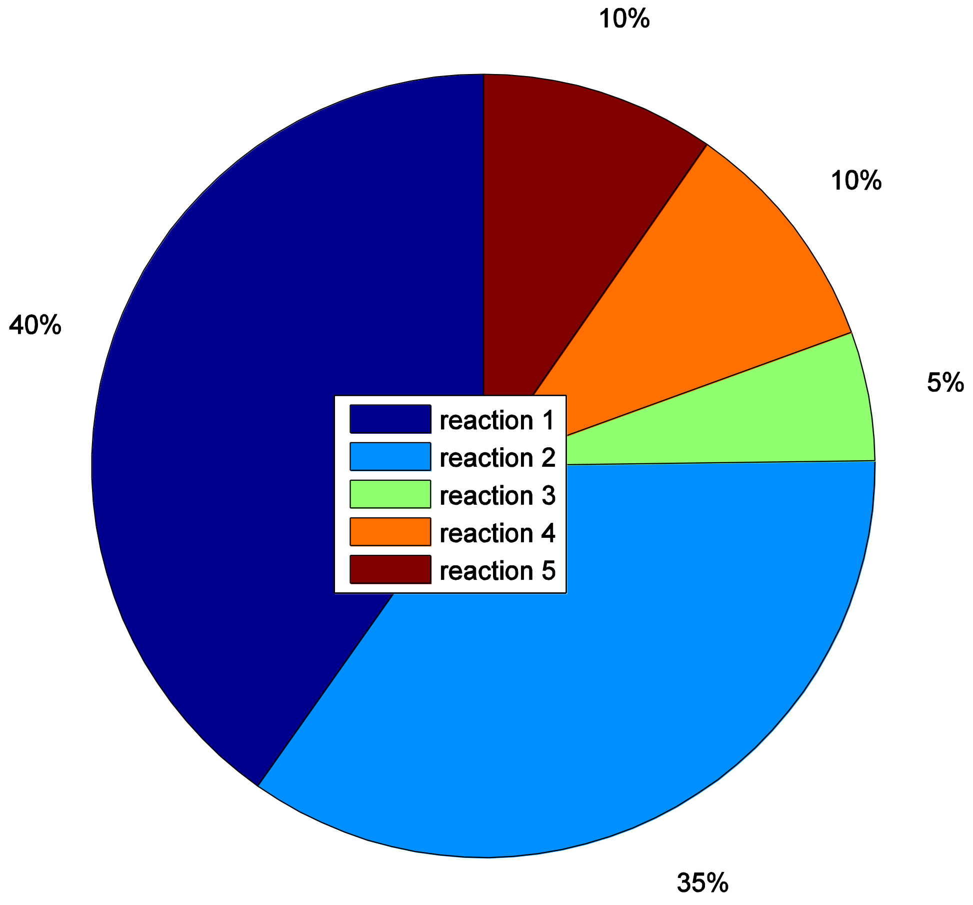

Figure S5. Steady-state distribution for binding energy around noise peaks. Colors represent the values of binding energy in the order of cyan, red, blue, magenta and green. The unit of binding energy is Kcal/mol.

noOL_OR3-r1

OR1	OR2
-10.39	-9.17
-10.40	-9.18
-10.41	-9.19
-10.42	-9.20
-10.43	-9.21

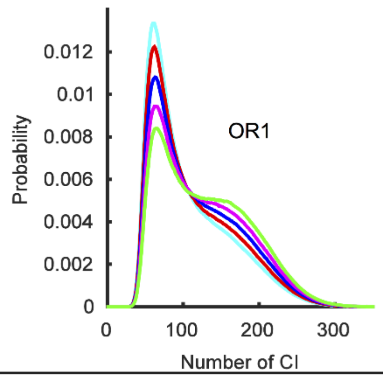

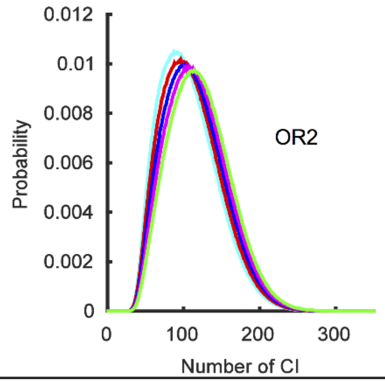

OR3-r1

OR1	OR2
-9.19	-7.96
-9.20	-7.97
-9.21	-7.98
-9.22	-7.99
-9.23	-8.00

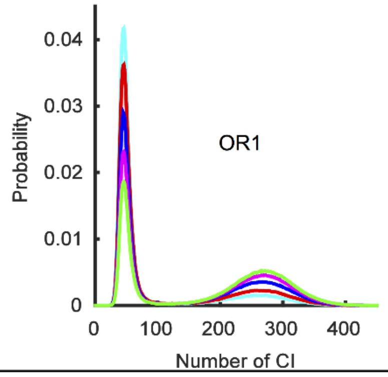

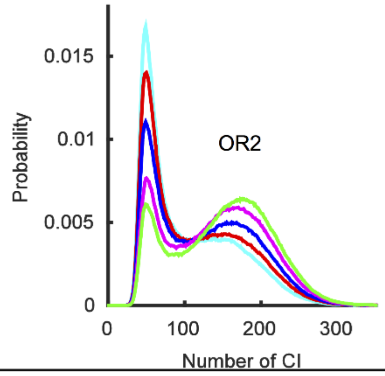

OL3-4

OR1	OR2
-9.38	-7.75
-9.39	-7.76
-9.40	-7.77
-9.41	-7.78
-9.42	-7.79

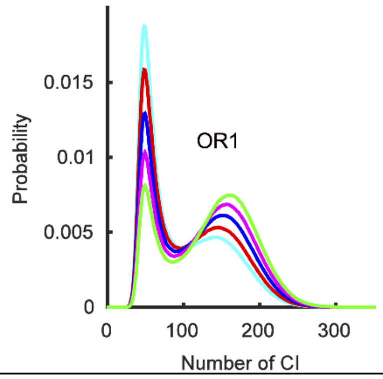

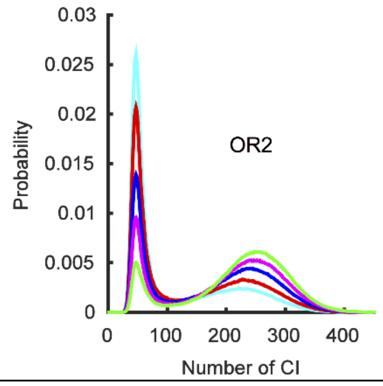

OL3-4_OR3-r1

OR1	OR2
-8.8	-7.72
-8.81	-7.73
-8.82	-7.74
-8.83	-7.75
-8.84	-7.76

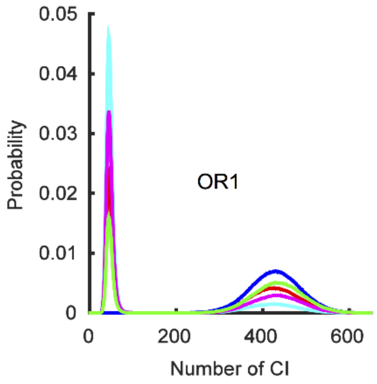

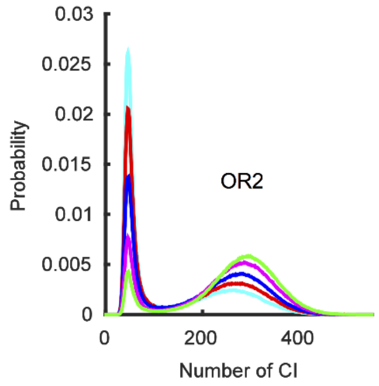

Table S1 Binding Configuration of 113 States. Black points in columns OL and OR refer to the situation that there are CI dimers bound there. Black points in columns c-OL and c_OR refer to the situation that corresponding coorparativity happens. Black points in columns 'Tetramer' and 'Octamer' refer to the situation that tetramer and/or octamer exists. Last three columns are AY's UP element activation model, CM's UP element activation model and AY's looped octamer model, respectively. The corresponding details of their activation groups are listed below.

Binding configuration	OL1	OL2	OL3	OR1	OR2	OR3	c-OL12	c-OL23	c-OR12	c-OR23	Tetramer	Octamer	AY-UP	CM-UP	AY-LO
1													unact	unact	unact
2	●												unact	unact	unact
3		●											unact	unact	unact
4	●	●					●						unact	unact	unact
5			●										unact	unact	unact
6	●		●										unact	unact	unact
7		●	●					●					unact	unact	unact
8	●	●	●				●						unact	unact	unact
9	●	●	●					●					unact	unact	unact
10				●									unact	unact	unact
11	●			●									unact	unact	unact
12		●		●									unact	unact	unact
13	●	●		●			●						unact	unact	unact
14			●	●									unact	unact	unact
15	●		●	●									unact	unact	unact
16		●	●	●				●					unact	unact	unact
17	●	●	●	●			●						unact	unact	unact
18	●	●	●	●				●					unact	unact	unact
19					●								act1	act1	act1
20	●				●								act1	act1	act1
21		●			●								act1	act1	act1
22	●	●			●		●						act1	act1	act1
23			●		●								act1	act1	act1
24	●		●		●								act1	act1	act1
25		●	●		●			●					act1	act1	act1
26	●	●	●		●		●						act1	act1	act1
27	●	●	●		●			●					act1	act1	act1
28				●	●				●				act1	act1	act1
29	●			●	●				●				act1	act1	act1
30		●		●	●				●				act1	act1	act1
31	●	●		●	●		●		●				act1	act1	act1
32			●	●	●				●				act1	act1	act1
33	●		●	●	●				●				act1	act1	act1
34		●	●	●	●			●	●				act1	act1	act1
35	●	●	●	●	●		●		●				act1	act1	act1
36	●	●	●	●	●			●	●				act1	act1	act1
37						●									
38	●					●									
39		●				●									
40	●	●				●	●								
41			●			●									
42	●		●			●									
43		●	●			●		●							
44	●	●	●			●	●								
45	●	●	●			●		●							
46				●		●									
47	●			●		●									
48		●		●		●									
49	●	●		●		●	●								
50			●	●		●									
51	●		●	●		●									
52		●	●	●		●		●							
53	●	●	●	●		●	●								
54	●	●	●	●		●		●							
55					●	●				●					
56	●				●	●				●					
57		●			●	●				●					
58	●	●			●	●	●			●					
59			●		●	●				●					
60	●		●		●	●				●					
61		●	●		●	●		●		●					
62	●	●	●		●	●	●			●					
63	●	●	●		●	●		●		●					
64				●	●	●			●						
65	●			●	●	●			●						
66		●		●	●	●			●						
67	●	●		●	●	●	●		●						
68			●	●	●	●			●						
69	●		●	●	●	●			●						
70		●	●	●	●	●		●	●						
71	●	●	●	●	●	●	●		●						
72	●	●	●	●	●	●		●	●						
73				●	●	●				●					
74	●			●	●	●				●					
75		●		●	●	●				●					
76	●	●		●	●	●	●			●					
77			●	●	●	●				●					
78	●		●	●	●	●				●					
79		●	●	●	●	●		●		●					
80	●	●	●	●	●	●	●			●					
81	●	●	●	●	●	●		●		●					
82	●	●		●	●		●		●			●	act2	act3	act2
83		●	●	●	●			●	●			●	act3	act2	act2
84	●	●	●	●	●			●	●			●	act3	act2	act2
85	●	●	●	●	●		●		●			●	act3	act2	act3
86	●	●		●	●		●		●			●	act1	act3	act2
87		●	●	●	●			●	●			●	act1	act2	act2
88	●	●	●	●	●			●	●			●	act1	act2	act3
89	●	●	●	●	●		●		●			●	act1	act2	act2
90	●	●			●	●	●			●		●			
91		●	●		●	●		●		●		●			
92	●	●	●		●	●		●		●		●			
93	●	●	●		●	●	●			●		●			
94	●	●			●	●	●			●		●			
95		●	●		●	●		●		●		●			
96	●	●	●		●	●		●		●		●			
97	●	●	●		●	●	●			●		●			
98	●	●		●	●	●	●		●			●			
99		●	●	●	●	●		●	●			●			
100	●	●	●	●	●	●		●	●			●			
101	●	●	●	●	●	●	●		●		●	●			
102	●	●		●	●	●	●		●			●			
103		●	●	●	●	●		●	●			●			
104	●	●	●	●	●	●		●	●		●	●			
105	●	●	●	●	●	●	●		●			●			
106	●	●		●	●	●	●			●		●			
107		●	●	●	●	●		●		●		●			
108	●	●	●	●	●	●		●		●	●	●			
109	●	●	●	●	●	●	●			●		●			
110	●	●		●	●	●	●			●		●			
111		●	●	●	●	●		●		●		●			
112	●	●	●	●	●	●		●		●		●			
113	●	●	●	●	●	●	●			●	●	●			

Table S2 Results of mean and noise level of five constructs in the two-step expression model, the constant cell growth model and the stochastic cell growth model, using AY's and CM's UP Model separately. CV% refers to the ratio of CV between theory and experiments in percentage. In result of stochastic cell growth model, CV of cell doubling time is set as 0.14. Parameters for A1, A2 and A3 in CM's UP Model are estimated as 1, 0.95, and 2.2, respectively.

AY's UP Model						
		WT	noOL_OR3-r1	OR3-r1	OL3-4	OL3-4_OR3-r1
Two-step	mean	162	301	389	476	625
	CV%	37.3%	59.4%	53.0%	55.3%	51.2%
	Fano	1.8	4.6	3.8	3.8	4.4
Ito_cc	mean	162	300	389	476	626
	CV%	41.2%	64.1%	57.4%	60.5%	55.2%
	Fano	2.2	5.4	4.4	4.5	5.1
SSA_cc	mean	161	301	389	476	625
	CV%	42.3%	64.7%	57.7%	59.8%	55.6%
	Fano	2.3	5.5	4.4	4.4	5.2
Ito_sc	mean	162	300	390	477	625
	CV%	47.1%	76.7%	71.5%	77.3%	76.0%
	Fano	2.8	7.7	6.9	7.4	9.6

CM's UP Model						
		WT	noOL_OR3-r1	OR3-r1	OL3-4	OL3-4_OR3-r1
Two-step	mean	154	301	345	476	625
	CV%	38.5%	59.6%	55.7%	55.6%	51.6%
	Fano	1.8	4.7	3.7	3.8	4.4
Ito_cc	mean	155	301	345	476	625
	CV%	42.1%	64.6%	60.4%	60.4%	56.1%
	Fano	2.2	5.5	4.3	4.5	5.2
SSA_cc	mean	154	301	345	476	624
	CV%	42.8%	64.7%	60.4%	59.7%	55.6%
	Fano	2.2	5.5	4.3	4.4	5.1
Ito_sc	mean	155	301	345	476	625
	CV%	47.4%	76.8%	73.4%	77.7%	76.1%
	Fano	2.7	7.8	6.4	7.4	9.6

Table S3 Six computational constructs for lower bounds of mean expression level and intrinsic noise strength. ΔGRx refers to the binding energy between ORx and CI.

Unit: Kcal/mol	Mean Expression Level	Intrinsic Noise Strength
$\Delta GR1 = -4.1$	43	0.80
$\Delta GR2 = -4.1$	41	0.68
$\Delta GR3 = -15$	42	0.68
$\Delta GR1 = -4.1$<br>$\Delta GR2 = -4.1$	41	0.67
$\Delta GR1 = -4.1$<br>$\Delta GR3 = -15$	41	0.68
$\Delta GR1 = -4.1$<br>$\Delta GR2 = -4.1$<br>$\Delta GR3 = -15$	41	0.67
